# Fucosyltransferase 8‐Derived Circular RNA Drives M2 Polarization of Macrophages Through ENO1‐TNF Signaling Axis to Promote Lung Cancer Progression

**DOI:** 10.1111/1759-7714.70194

**Published:** 2025-12-02

**Authors:** Yang Ren, Yidan Shen, Qingguo Wu, Peng Zhang, Lei Wang, Feng Li, Yinzhong Shen

**Affiliations:** ^1^ Department of Respiratory Disease and Critical Care Medicine, Shanghai Public Health Clinical Center Fudan University Shanghai China; ^2^ Shanghai Institute of Infectious Disease and Biosecurity Fudan University Shanghai China; ^3^ Department of Infection and Immunity, Shanghai Public Health Clinical Center Fudan University Shanghai China

**Keywords:** circFUT8, ENO1, lung cancer, macrophages

## Abstract

**Background:**

Lung cancer, representing a predominant form of pulmonary malignancy, demonstrates significant disease burden and poor clinical outcomes. Circular RNAs (circRNAs) have emerged as critical regulators in various cancers, including lung cancer. However, the specific roles and mechanisms of circRNAs in lung cancer remain largely unexplored.

**Methods:**

Differential circRNA expression was analyzed using GEO datasets GSE101586 and GSE112214. CircFUT8 was prioritized for its upregulation in lung cancer tissues. In vitro and in vivo functional experiments evaluated its effects on cell proliferation, apoptosis, migration, and invasion. RNA pull‐down, immunofluorescence, and western blotting assessed interactions with ENO1. Macrophage polarization was examined via cocultures and flow cytometry.

**Results:**

CircFUT8 (hsa_circ_0003028) was significantly upregulated in lung cancer tissues, correlating with advanced stages and poor prognosis. It enhanced lung cancer cell proliferation, migration, and invasion while inhibiting apoptosis in cellular and animal models. Mechanistically, circFUT8 directly binds ENO1 to form an RNA‐protein complex, promoting M2 macrophage polarization. Silencing circFUT8 reversed these effects by suppressing ENO1 and M2 polarization, inhibiting tumor progression. Moreover, ENO1 promotes TNF signaling through glycolytic metabolites.

**Conclusions:**

Our findings highlight the critical role of circFUT8 in lung cancer progression through its regulation of M2 macrophage polarization via interaction with ENO1. The findings suggest that circFUT8 may serve as both a diagnostic marker and a promising therapeutic target in lung cancer management. This study first identified the regulating oncogenic role of circFUT8 in lung cancer progression and the microenvironment.

## Graphical abstract

The novel mechanism by which circFUT8 promotes lung cancer progression is that circFUT8 binds to ENO1, enhancing glycolytic metabolism to the upregulation of JUN, CSF2, CSF1, TRAF1, CCL5, MMP9, which in turn drive the polarization of tumor‐associated macrophages toward an immunosuppressive M2 phenotype, fostering a pro‐tumor microenvironment.

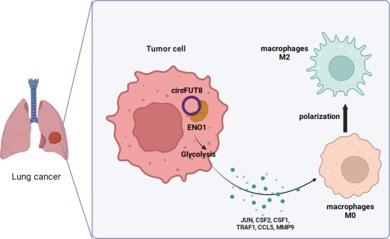

## 1 Introduction

Lung cancer is a leading cause of cancer‐related mortality, with non‐small cell lung cancer (NSCLC) comprising approximately 85% of all lung cancer cases [1]. Within NSCLC, lung adenocarcinoma (LUAD) is the most common subtype, representing about 60% of NSCLC cases [2, 3]. LUAD often lacks distinctive clinical features, leading to a high recurrence rate and low survival rate [4]. In contrast, LUSC is more prevalent among smokers and has a poor prognosis despite advances in treatment. Currently, surgical interventions, chemoradiotherapy, targeted therapies, and immunotherapy have been implemented to improve patient outcomes in LUAD patients [5, 6]. Despite these advancements, therapeutic efficacy remains suboptimal [7]. The development of novel diagnostic markers and treatment targets is crucial for enhancing disease management and optimizing patient prognosis [8].

Circular RNAs (circRNAs) are a unique class of noncoding RNAs formed by covalently closed loops through back‐splicing events. Unlike linear RNAs, circRNAs are highly stable due to their circular structure, which resists exonucleolytic degradation [9, 10]. Researchers have identified circRNAs with significantly altered expression levels in lung cancer tissue [11, 12]. In lung cancer, circRNAs are emerging as oncodrivers/tumor suppressors and promising biomarkers [9, 13, 14], by regulating cell proliferation, apoptosis, invasion, and metastasis, thereby influencing the progression of lung cancer [15, 16]. Mechanistically, circRNAs can act as miRNA sponges, binding to proteins, and regulating gene transcription and splicing processes [14, 17]. Given the significant role of circRNAs in lung cancer, they are considered potential therapeutic targets.

Notably, circRNAs play regulatory roles in shaping the tumor microenvironment (TME) characteristics, showing promise for enhancing the efficiency and outcomes of immunotherapeutic regimens [18, 19, 20]. Tumor‐associated macrophages (TAMs) serve as key immunosuppressive regulators influencing cancer progression [21]. While M1‐polarized macrophages function as pro‐inflammatory mediators, their M2 counterparts primarily contribute to malignant growth [22]. Higher M1 macrophage infiltration correlates with improved patient outcomes [23]. Conversely, elevated M2 TAM populations are linked to unfavorable clinical prognoses. Studies have shown that M2‐type TAMs contribute to poor prognosis [24]. Studies show that circRNAs regulate TAM polarization in colorectal cancer [25, 26], breast cancer [27], and other cancer types [28, 29, 30], and their function in LUAD TAM reprogramming remains largely unknown [21, 31]. Therefore, exploring the role of circRNAs underlying TAMs macrophages M2 polarization would provide novel perspectives for uncovering the complexities of lung cancer progression.

Here, through comprehensive analysis of transcriptomic profiles, we identified a Fucosyltransferase 8 derived circRNA (circFUT8, hsa_circ_0003028) upregulated in tumor specimens and showing clinical relevance in lung cancer. Functionally, circFUT8 enhances cellular growth, motility, and invasive potential, while suppressing programmed cell death in vitro and in vivo. Mechanistically, circFUT8 facilitates the M2 polarization of TAMs by interacting with ENO1 in the tumor cells. This study identified the oncogenic role of circFUT8 in lung cancer progression and microenvironment.

## 2 Materials and Methods

### 2.1 CircRNA Expression Analysis

The GEO datasets GSE101586 [32] and GSE112214 (https://www.ncbi.nlm.nih.gov/geo/query/acc.cgi?acc=GSE112214) were systematically analyzed to reveal dysregulated circRNAs in patients with lung cancer. The raw RNA‐seq data generated from the circFUT8 knockdown experiments in A549 and H1299 cells for this study have been deposited in the NCBI Gene Expression Omnibus (GEO) under accession number GSE307293.

### 2.2 Clinical Specimens

This study included 25 paired lung cancer tumor and normal tissue. All samples were collected at the time of surgical resection and were rapidly frozen in liquid nitrogen for subsequent analysis. The clinical information of the patients, including tumor stage (TNM classification), and tumor size (T stage), was meticulously recorded and organized from medical records for further analysis. The levels of circFUT8 in both cancerous and normal tissues were measured via qRT‐PCR. The association between circFUT8 levels and lung cancer patient outcomes was evaluated using Kaplan–Meier analysis. Patients were categorized into high and low‐expression groups based on circFUT8 levels, and their survival rates were compared using the Log‐rank test. Survival duration was tracked from surgery until death or the final follow‐up. A post hoc power analysis was performed using G*Power 3.1, which indicated that the sample size of 25 paired tissues provided a statistical power of > 0.90 to detect a significant difference in circFUT8 expression (effect size *d* = 1.2, *α* = 0.05, two‐tailed *t*‐test), supporting the adequacy of the sample size for the primary comparative analysis.

### 2.3 Cell Culture

The human lung cancer cell lines (A549 and H1299), as well as HEK‐293T and THP‐1 cells, were sourced from the Chinese Academy of Sciences Cell Resource Center. These cells were cultured in DMEM or RPMI‐1640 medium (Gibco, USA), supplemented with 10% fetal bovine serum (Gibco, USA), and maintained under standard conditions (37°C, 5% CO_2_).

### 2.4 Treatment With RNase R and RNase A

To distinguish circRNAs from their linear counterparts, RNA samples were subjected to enzymatic digestion using RNase R (Thermo Fisher, USA), which selectively degrades linear RNA while preserving circRNA structures. Subsequently, RNase A treatment was performed to specifically cleave circRNA molecules. For each reaction, 2 μg of RNA was treated with 10 units of the respective enzyme for 30 min at 37°C. The processed RNA was then extracted using the TRIeasy Plus total RNA isolation kit (Yeasen, Shanghai) and subjected to quantitative reverse transcription PCR analysis.

### 2.5 Reverse Transcription Quantitative PCR

RNA was extracted from both cell cultures and tumor tissues using TRIzol reagent (R0011, Beyotime, China), adhering to the supplier's guidelines. The purity and quantity of RNA were determined with the Nano‐500 microspectrophotometer (Allsheng, Hangzhou, China). For cDNA synthesis, 1 μg of RNA was processed with the Script Reverse Transcription Reagent Kit (6110A, TaKaRa, Japan). Quantitative PCR was conducted using the SYBR Premix Ex Taq II Kit (RR820B, TaKaRa) on the ABI StepOne Real‐time Detection System (LTC, Carlsbad, USA). The thermal cycling protocol included an initial denaturation at 95°C for 5 min, followed by 40 cycles of 95°C for 15 s and 60°C for 45 s. Primer sequences used in the study are detailed in Table 1. Expression levels of target genes were referenced to GAPDH and quantified using the 2^−ΔΔCt^ method.

**TABLE 1:** The primers for RT‐qPCR.

Gene name	Forward primer (5′‐3′)	Reverse primer (5′‐3′)
Circ‐FUT8	5′‐CAGGACTCCAGGGAAGTGAG‐3′	5′‐ATCTTGGACAGTTCTCGGCT‐3′
GAPDH	5′‐GGAGCGAGATCCCTCCAAAAT‐3′	5′‐GGCTGTTGTCATACTTCTCATGG‐3′
JUN	5′‐TCCAAGTGCCGAAAAAGGAAG‐3′	5′‐CGAGTTCTGAGCTTTCAAGGT‐3′
CSF2	5′‐TCCTGAACCTGAGTAGAGACAC‐3′	5′‐TGCTGCTTGTAGTGGCTGG‐3′
CSF1	5′‐TGGCGAGCAGGAGTATCAC‐3′	5′‐AGGTCTCCATCTGACTGTCAAT‐3′
TRAF1	5′‐TCCTGTGGAAGATCACCAATGT‐3′	5′‐GCAGGCACAACTTGTAGCC‐3′
CCL5	5′‐CCAGCAGTCGTCTTTGTCAC‐3′	5′‐CTCTGGGTTGGCACACACTT‐3′
MMP9	5′‐TGTACCGCTATGGTTACACTCG‐3′	5′‐GGCAGGGACAGTTGCTTCT‐3′
IL‐1β	5′‐ATGATGGCTTATTACAGTGGCAA‐3′	5′‐GTCGGAGATTCGTAGCTGGA‐3′
CCL‐20	5′‐TGCTGTACCAAGAGTTTGCTC‐3′	5′‐CGCACACAGACAACTTTTTCTTT‐3′
IL‐6	5′‐CCCACCGGGAACGAAAGAGA‐3′	5′‐TTCTCCTGGGGGTATTGTGGAG‐3′
Arg‐1	5′‐GTGGAAACTTGCATGGACAAC‐3′	5′‐AATCCTGGCACATCGGGAATC‐3′

### 2.6 Cellular Localization Analysis Using FISH and Immunofluorescence

For subcellular localization studies of circFUT8 and ENO1, lung cancer cells were fixed using 4% paraformaldehyde, followed by prehybridization and overnight hybridization (37°C) with fluorophore‐conjugated circFUT8 probes. After blocking with goat serum (30 min), samples were treated with primary antibodies (1 h) and subsequently with Alexa Fluor 488‐labeled secondary antibodies (Yeasen, Shanghai) for 1 h at room temperature. Following PBS washes, nuclei were counterstained with DAPI‐containing mounting medium, and fluorescent images were acquired using microscopy. For tissue‐based FISH analysis, four distinct regions from each TMA spot were selected for circFUT8 quantification. The Image‐Pro Plus software (Media Cybernetics, USA) was employed to measure optical density, with the average intensity from four regions representing the circFUT8 expression level in each sample.

### 2.7 Cell Transfection

All recombinant plasmids were synthesized and validated by Genomeditech (Shanghai, China). Lentiviral particles were generated using a commercial packaging system (Yeasen, Shanghai) and introduced into target cells via liposome‐mediated transfection. For circFUT8 silencing experiments, A549 and H1299 cells were transduced with lentiviral vectors encoding either shRNA targeting circFUT8 (sh‐circFUT8) or non‐targeting control shRNA (sh‐NC). To establish ENO1‐overexpressing cell lines, lung cancer cells were infected with lentiviral particles containing the ENO1 expression construct (oe‐ENO1). Additionally, lentiviruses carrying empty plasmids (vector) served as the control group. The above lentiviruses were obtained from GenePharma (China). Cells were seeded into T25 culture flasks individually and cultured overnight. Then, 2 mL of culture medium without penicillin/streptomycin was added to each bottle. Lentivirus was directly added to the culture medium. Following overnight incubation (16 h), the lentiviral supernatant was removed and substituted with fresh complete medium. Stable transfectants were selected through puromycin treatment (1 μg/mL) for 7 days, commencing 72 h post‐transduction.

### 2.8 Cell Proliferation Assessment Using CCK‐8

Transfected A549 and H1299 cells were seeded at a density of 1 × 10^3^ cells per well in a 96‐well plate, with at least three replicates per sample. After incubation with 10 μL CCK‐8 reagent (Beyotime) at 37°C for 2 h, the cells were divided into four groups: A549 and H1299 cells. Absorbance was measured at 450 nm using a microplate spectrophotometer (Thermo Fisher Scientific) at 2, 24, 48, and 72 h after cell attachment.

### 2.9 Cell Apoptosis and Surface Marker Analysis by Flow Cytometry

For apoptosis detection, approximately 2 × 10^6^ cells were harvested and stained with Annexin V‐FITC (10 μL; C1062S‐1, Beyotime) and propidium iodide (5 μL; C1062S‐3, Beyotime) in dark conditions for 10 min, followed by immediate flow cytometric analysis.

For macrophage polarization studies, PMA‐stimulated THP‐1 cells were cocultured with sh‐circFUT8‐transfected A549 or H1299 cells. Surface marker expression was evaluated by incubating cells with FITC‐labeled anti‐CD206 and PE‐conjugated anti‐CD86 antibodies in PBS (30 min, 4°C), followed by analysis using a BD Biosciences flow cytometer.

### 2.10 Cell Migration Assay

The four groups of transfected cells (A549‐NC, A549 sh‐circFUT8, H1299, and H1299 sh‐circFUT8) were plated in a six‐well plate. Before plating, a parallel line was drawn on the bottom of the six‐well plate and marked, and then an appropriate number of cells were spread in each well. On the next day, when the cell density confluence reached 80%–90%, the medium was discarded, and 200 μL of the tip of the bullet was used to draw lines on the bottom of the dish to ensure that the lines were perpendicular to the marking line on the bottom of the six‐well plate. Then the cells were rinsed with PBS to wash out the loose adherent and floating cells, so as not to influence the photo taking. Subsequently, photos were taken under the microscope at 0, 24, 36, and 48 h. Subsequently, the cell migration rate was calculated according to the migration rate = (initial distance − final distance)/initial distance or (initial area − final area)/initial area, and the cell migration ability of each group was evaluated.

### 2.11 Cell Invasion Assessment Using Transwell System

Cell invasive potential was evaluated using Matrigel‐coated transwell inserts (24‐well format; Corning, USA). Remove the matrix glue from the −20°C refrigerator and place it at 4°C for dissolution. At the same time, pre‐cool the sterile gun tip at −20°C in advance. After the matrix glue is dissolved, take the sterile 24‐well plate and the chamber, and carry out aseptic operation on the ultra‐clean table. After that, the 24‐well plates coated with Matrigel were placed in the cell incubator for 4 h for subsequent use. The transfected lung cancer cells A549‐NC, A549 sh‐circFUT8, H1299, and H1299 sh‐circFUT8, and H1299‐shcircFUT8 were counted, and then 200 μL serum‐free medium containing 5 × 10^4^ cells was added to each well of the chamber. 500 μL of complete medium (RPMI + 10% FBS) was added to the bottom of the 24‐well plate chamber, then placed into the cell incubator and incubated for 24–48 h, with at least 6 secondary wells per sample. The 24‐well plates were removed from the incubator at 24, 36, and 48 h, respectively. After 24 h, one of the wells of each sample was stained with crystal violet. First, the medium above the chamber was removed with a pipette, then rinsed with PBS, and 600 μL crystal violet was added to the 24‐well plate. The chamber was placed on a slide, and the number of cells in the chamber was observed under a microscope to further determine whether the cells had completed the invasion. The above operations were repeated at 36 and 48 h of culture, respectively, and crystal violet staining was used to further evaluate the cell invasion at the most appropriate time.

### 2.12 TAM Co‐Culture System

A transwell‐based co‐culture platform (Corning, USA) was established to investigate tumor cell–macrophage interactions. THP‐1 cells were seeded in the lower chamber and differentiated into macrophages by treatment with 100 ng/mL 12‐O‐tetradecanoylphorbol‐13‐acetate (PMA, 16561‐29‐8, Sigma‐Aldrich) for 48 h, while lung cancer cells were placed in the upper chamber. After PMA induction, the THP‐1‐derived macrophages were cocultured with A549 or H1299 cells transfected with the specified constructs.

### 2.13 Xenograft Nude Mice Model

Male C57BL/6 mice nude mice (5 weeks old) were randomly allocated into four experimental groups (*n* = 5 per group) using a blinded study design. Lentivirus‐transduced A549 and H1299 cells (1 × 10^6^ cells/mouse) expressing either sh‐circFUT8 or negative control constructs were subcutaneously injected into the left axillary region. Tumor growth was monitored by caliper measurements every 7 days for 21 days. Following euthanasia, xenograft tissues were harvested for subsequent immunohistochemical analysis, gene expression profiling, and protein detection.

### 2.14 RNA‐Protein Interaction Analysis

RNA‐associated protein complexes were isolated using the BersinBio RNA antisense purification system (Guangzhou, China). Cellular lysates were prepared from 2 × 10^7^ cells in buffer containing protease and RNase inhibitors, followed by centrifugation (16 000×*g*, 10 min). The resulting supernatant was incubated with biotin‐labeled circFUT8 probes (40 pmol) at 37°C for 3 h. Streptavidin‐coated magnetic beads were washed and subsequently mixed with the probe‐bound complexes for 30 min at room temperature. Captured proteins were eluted for subsequent identification by mass spectrometry and validation through immunoblotting analysis.

### 2.15 Western Blot

Protein extraction from cellular and tissue specimens was conducted using RIPA lysis buffer (Beyotime), with protein concentrations determined via a BCA assay kit (Thermo Fisher Scientific). Proteins (30 μg per lane) were separated on 10% SDS‐PAGE gels and transferred to PVDF membranes using a semi‐dry transfer system. Membranes were blocked with 5% non‐fat milk for 1 h at room temperature, followed by incubation with primary antibodies at 4°C overnight. After three TBST washes, membranes were exposed to horseradish peroxidase‐conjugated secondary antibodies (1:10 000; ab181236, Abcam, UK). Enhanced chemiluminescence substrate (P0018S, Beyotime) was used for band visualization, and quantification was performed using ImageJ software (NIH, USA).

### 2.16 Immunohistochemistry (IHC) Staining

Paraffin‐embedded xenograft tissue sections were subjected to immunohistochemical staining using specific primary antibodies targeting Ki67 and Cleaved‐caspase3. Antibody binding was detected using appropriate visualization methods, and stained sections were examined under an Olympus BX51 multifunctional microscope (Tokyo, Japan). Three independent pathologists, blinded to experimental conditions, conducted quantitative assessments using standardized evaluation criteria. The expression levels of Ki‐67 and Cleaved‐caspase3 were quantified using ImageJ software (NIH, USA). Five random fields of view (200× magnification) were captured per sample. For Ki‐67 (nuclear staining), the percentage of positive cells (Labeling Index) was calculated. For Cleaved‐caspase3 (cytoplasmic/nuclear staining), both the staining intensity (scored on a scale of 0–3: 0 = negative, 1 = weak, 2 = moderate, 3 = strong) and the percentage of positive cells were evaluated to generate an *H*‐score (range 0–300) using the formula: *H*‐score = (percentage of weak intensity cells × 1) + (percentage of moderate intensity cells × 2) + (percentage of strong intensity cells × 3).

### 2.17 Immunofluorescence (IF) Staining

Paraffin sections were deparaffinized with xylene, hydrated with gradient ethanol, and then antigen repair was performed with sodium citrate buffer (95°C, 15 min). Blocking solution containing 5% homologous serum + 1% BSA + 0.1% Tween‐20/PBS was dropped and blocked at room temperature for 1 h. Subsequently, CD206 antibody was incubated at 4°C overnight, washed three times with PBS/Tween‐20, and incubated with fluorescent secondary antibody for 1 h at room temperature in the dark. After washing with PBS, prehybridization buffer (50% formamide + 2 × SSC) was added and pretreated at 37°C for 1 h. FISH probe (10–50 nM hybridization solution) was added, and coverslips were used for hybridization. They were washed in 2 × SSC (50°C, 3 × 5 min) and 1 × SSC (room temperature, 2 × 5 min) containing formamide. The nuclei were stained with DAPI (1 μg/mL) in the dark for 5 min and washed with PBS for three times. Finally, the tablets were sealed with anti‐quenching sealant, sealed with nail polish, and dried in the dark before confocal microscopy imaging analysis. The fluorescence intensity of CD206 was quantified using ImageJ software. Five random fields of view (400× magnification) were captured for each sample under identical exposure settings. The mean fluorescence intensity (MFI) was measured after subtracting the background signal from adjacent non‐reactive areas. The nuclei (DAPI+) were used to count the total number of cells.

### 2.18 Lactate and ATP Measurement

Lactate levels in the cell culture supernatant were quantified using a Lactate Assay Kit (colorimetric, e.g., Sigma‐Aldrich, MAK064) according to the manufacturer's instructions. Briefly, supernatant was collected from cells cultured for 24 h under standard conditions. The reaction mixture was incubated at room temperature for 30 min, and the absorbance was measured at 570 nm. Intracellular ATP levels were measured using an ATP Assay Kit (Fluorometric, e.g., Abcam, ab83355). Cells were lysed, and the lysate was mixed with the reaction mix. Luminescence was measured immediately using a microplate reader. Values were normalized to the cell count.

### 2.19 Exogenous Lactate Treatment

For lactate rescue experiments, sodium lactate (Sigma‐Aldrich, L7022) was dissolved in PBS to create a stock solution. The circFUT8‐knockdown cells and corresponding controls were treated with a final concentration of 10 mM sodium lactate for 24 h before RNA extraction and qPCR analysis. An equivalent volume of PBS was added to the control groups.

### 2.20 Chromatin Immunoprecipitation (ChIP) Assay

The ChIP assay was performed using the SimpleChIP Plus Enzymatic Chromatin IP Kit (Magnetic Beads, Cell Signaling Technology, #9005) following the manufacturer's protocol. Briefly, A549 and H1299 cells were cross‐linked with 1% formaldehyde. Chromatin was digested enzymatically and immunoprecipitated overnight at 4°C with 5 μg of anti‐ENO1 antibody (Proteintech, 11204‐1‐AP) or normal rabbit IgG (negative control). Protein‐DNA complexes were eluted, and cross‐links were reversed. The purified DNA was analyzed by qPCR using primers specific for the promoter regions of the target genes.

### 2.21 Statistical Analysis

All statistical analyses were performed using GraphPad Prism 9.5.1. Differences between two groups were analyzed using the unpaired Student's *t*‐test, while comparisons involving three or more groups were conducted using one‐way ANOVA. A *p*‐value < 0.05 was considered statistically significant.

## 3 Results

### 3.1 Characteristics of circFUT8 in Lung Cancer

**FIGURE 1:**
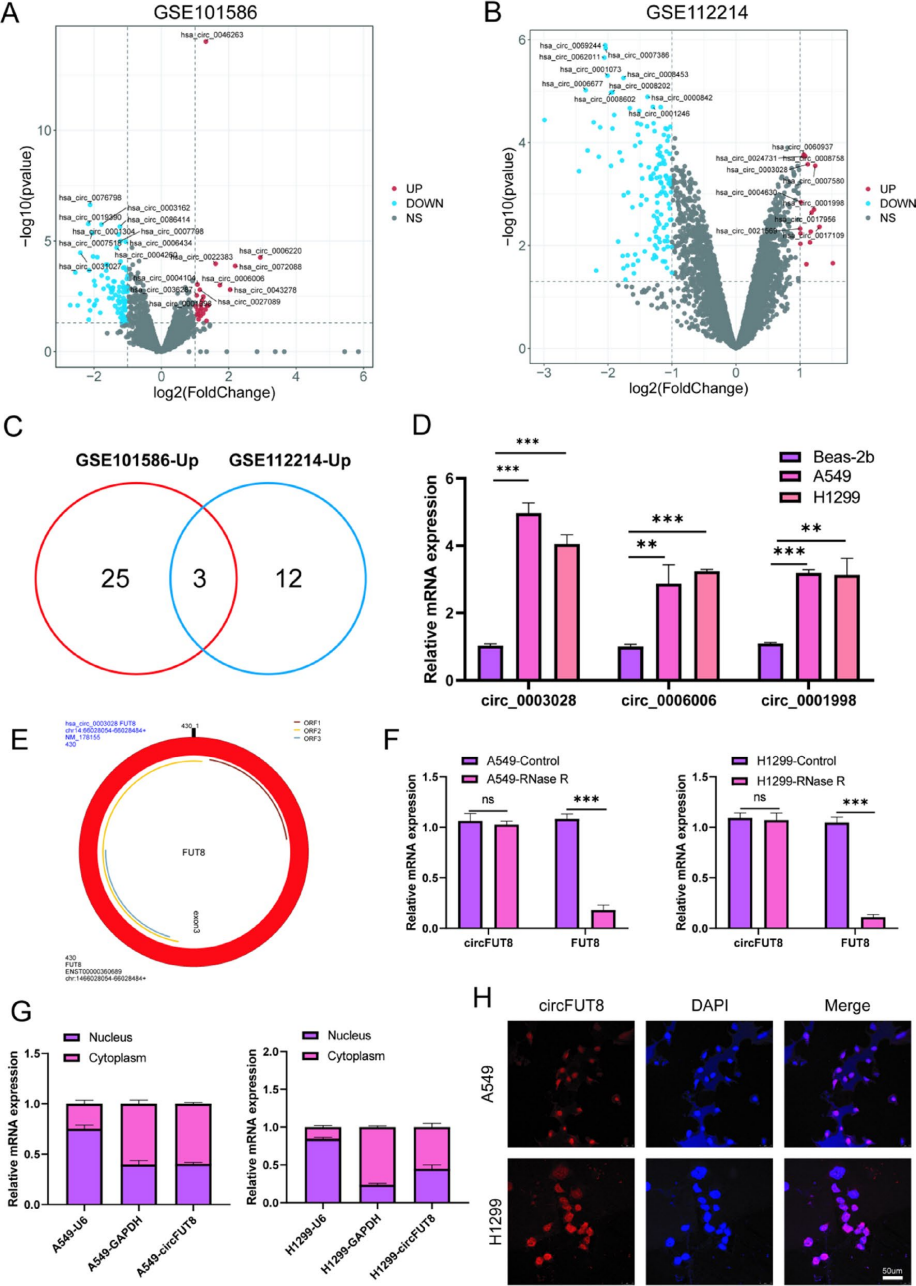
CircFUT8 is over expressed in lung cancer. (A) Differential expression analysis revealed 124 significantly altered circular RNAs across five matched lung cancer specimens using Arraystar microarray, with criteria at |log2FC| > 1.0 and *p* < 0.05. (B) Microarray profiling identified 148 circRNAs with significant expression changes in three paired lung adenocarcinoma samples, applying identical statistical thresholds (|log2FC| > 1.0, and *p* < 0.05). (C) Comparative analysis of upregulated circular RNAs between datasets GSE112214 and GSE101586 revealed overlapping candidate CircRNAs. (D) Quantitative reverse transcription PCR analysis demonstrated circFUT8 expression patterns in non‐small cell lung cancer cell lines. (E) Genomic characterization confirmed circ_0003028 from its host gene FUT8 through back‐splicing events. (F) RNase R resistance assays confirmed the circular nature of circFUT8 compared to its linear counterpart in A549 and H1299 cell lines. (G) Subcellular fractionation followed by qRT‐PCR analysis revealed distinct localization patterns of circFUT8, GAPDH, and U6 in cytoplasmic and nuclear compartments of lung cancer cells. (H) Fluorescence in situ hybridization analysis demonstrated predominant cytoplasmic localization of circFUT8 in both A549 and H1299 cell lines (scale bars: 50 μm). **p < 0.01, ***p < 0.001.

Recent investigations have identified numerous circRNAs exhibiting significant expression alterations between lung cancer tissues and their normal counterparts. CircRNA expression profiles were analyzed in GEO datasets from GSE101586 and GSE112214. The GSE101586 dataset revealed 124 circRNAs with significant expression changes, including 28 upregulated circRNAs and 96 downregulated circRNAs. The differential expression landscape of circRNAs in lung cancer is visualized through a volcano plot (Figure 1A), accompanied by a quantitative comparison of their expression levels in three paired normal and tumor tissue samples. Next, a total of 148 differentially expressed circRNAs was identified in GSE 112214, including 15 upregulated circRNAs and 135 downregulated circRNAs (Figure 1B). A Venn diagram displays that three common upregulated circRNAs were identified between GSE101586 and GSE112214 (Figure 1C).

From the intersection of GSE101586 and GSE112214 datasets, three consistently upregulated circRNAs exhibiting significant expression alterations were selected for further validation. Quantitative analysis revealed that hsa_circ_0003028 displayed the most substantial upregulation in lung cancer cell lines (A549 and H1299) relative to normal bronchial epithelial cells (BEAS‐2B) (Figure 1D). Based on these findings, we proceeded to characterize the biological properties of hsa_circ_0003028. hsa_circ_0003028 (also known as circFUT8) was matched to host genes. hsa_circ_0003028 is back‐spliced from 1 exon (exons3) of the FUT8 gene (Figure 1E). The RNase R digestion assay revealed that FUT8 levels were significantly reduced in A549 and H1299 cells following RNase R treatment, whereas the abundance of circ_0003028 remained stable (Figure 1F). Additionally, the subcellular localization of circFUT8 was predominantly cytoplasmic, as demonstrated by RNA FISH and nucleo‐cytoplasmic separation experiments (Figure 1G,H).

### 3.2 Prognostic Significance of circFUT8 in Lung Cancer

**FIGURE 2:**
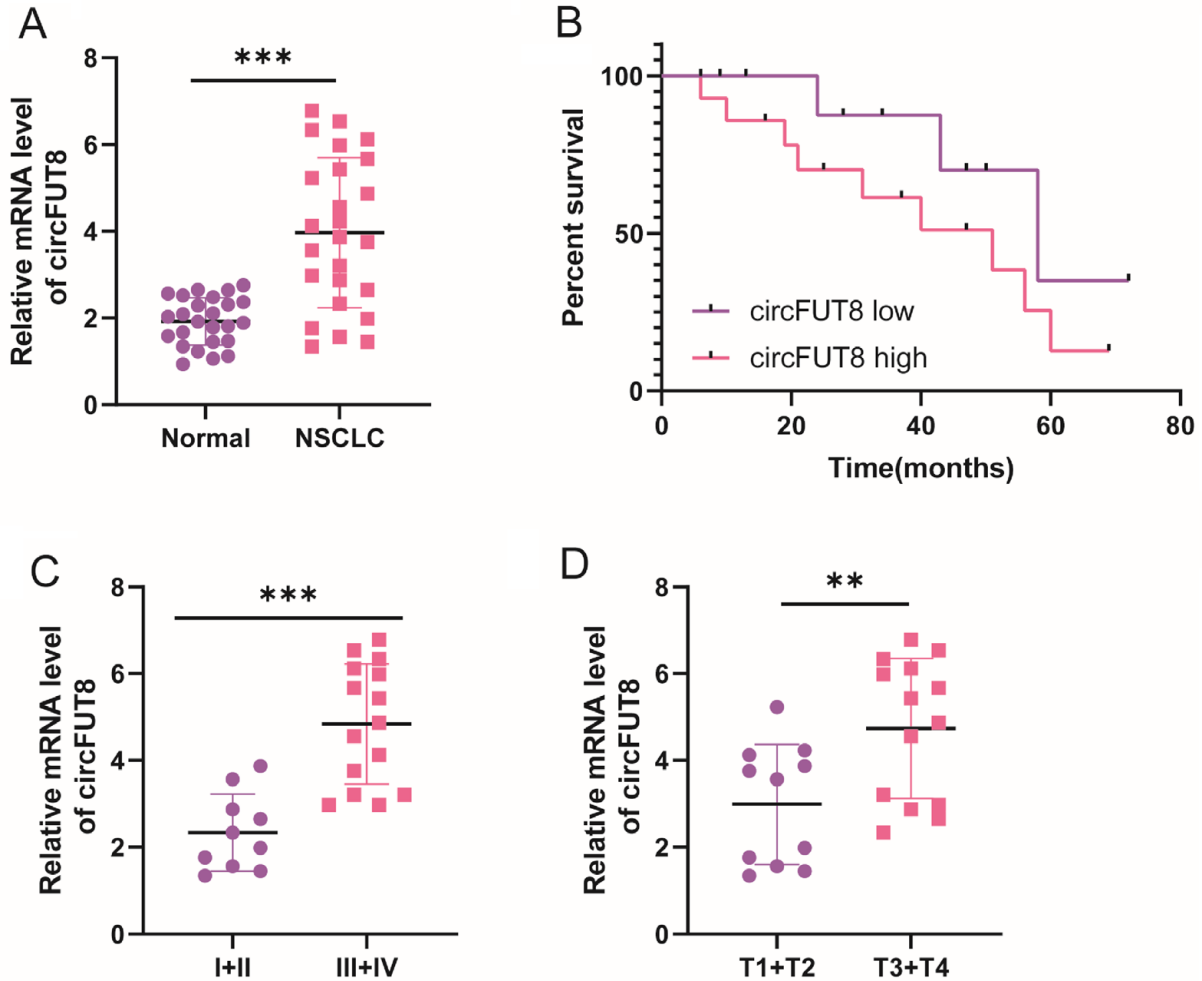
The prognostic significance of circFUT8 overexpression in lung cancer carcinoma. (A) Quantitative reverse transcription PCR analysis comparing circFUT8 expression levels between malignant and adjacent normal tissues from 25 NSCLC patients (mean ± SD). (B) Survival analysis using Kaplan–Meier curves illustrating the correlation between circFUT8 expression levels (high vs. low) and overall survival rates in NSCLC patients. (C) The expression of circFUT8 in TNM stage I + II and TNM stage III + IV of NSCLC patient. (D) The expression of circFUT8 in T1 + T2 stage and T3 + T4 stage of NSCLC patients. **p < 0.01, ***p < 0.001.

The clinical relevance of circFUT8 was evaluated in a cohort of 25 lung cancer patients, whose demographic and pathological characteristics are detailed in Figure 2. Comparative analysis revealed significant upregulation of circFUT8 in malignant tissues relative to adjacent normal samples (*n* = 25) (Figure 2A). Survival analysis demonstrated that elevated circFUT8 expression was associated with unfavorable clinical outcomes in lung cancer patients (Figure 2B). Furthermore, circFUT8 levels were significantly increased in advanced‐stage (III + IV) compared to early‐stage (I + II) tumors (Figure 2C). A similar trend was observed when comparing T2 + T4 stage tumors with T1 + T2 stage specimens (Figure 2D).

### 3.3 CircFUT8 Silencing Suppresses Malignant Phenotypes of Lung Cancer

**FIGURE 3:**
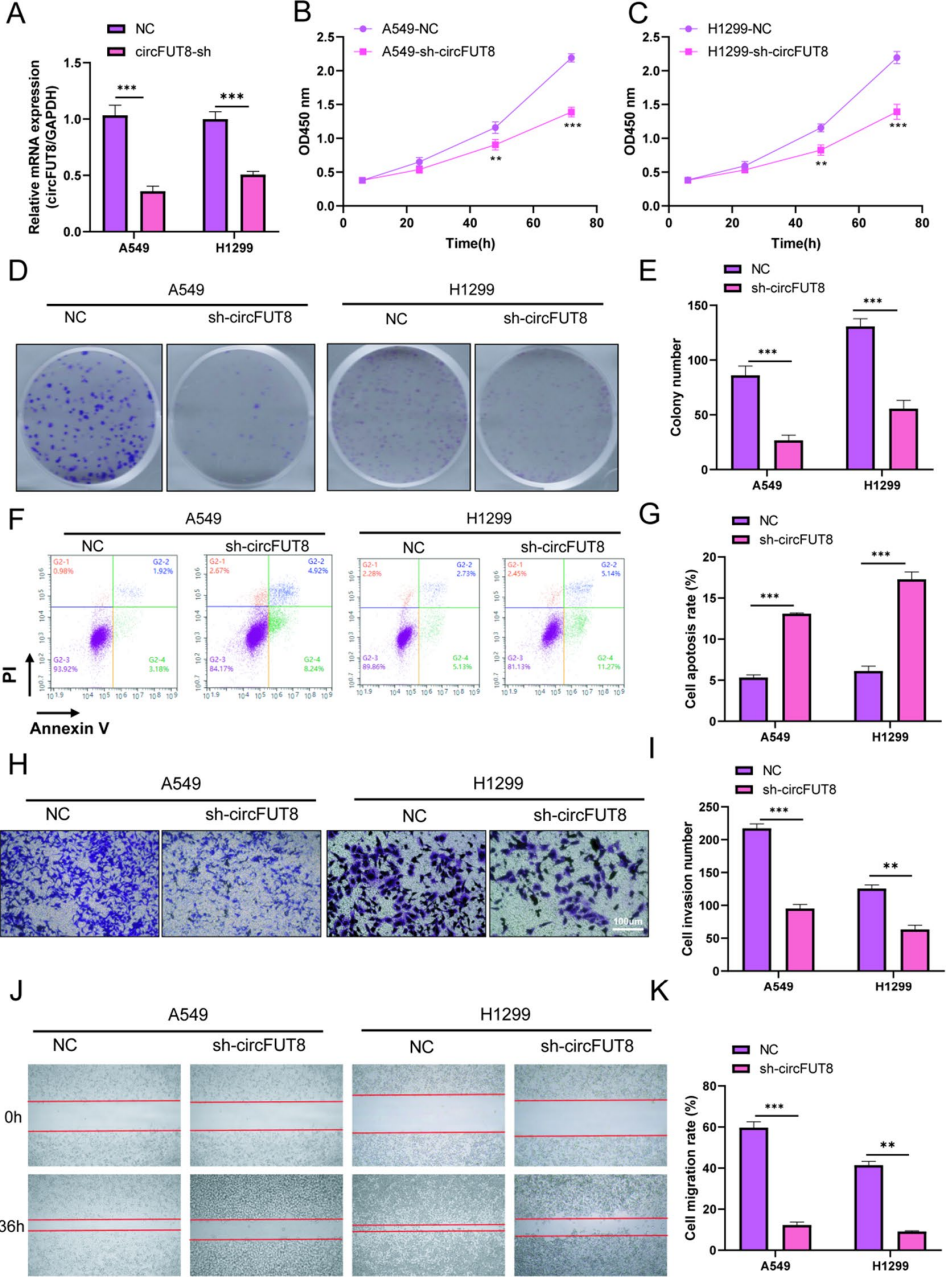
CircFUT8 knockdown exerts tumor‐suppressive effects in lung cancer cells. (A) CircFUT8 expression was determined using RT‐qPCR. (B) A549 cell viability was evaluated by CCK‐8. (C) H1299 cell viability was evaluated by CCK‐8. (D, E) Cell proliferation was evaluated by clone formation assay. (F, G) Cell apoptosis was examined using flow cytometry and Annexin V/PI staining. (H, I) Cell invasion was evaluated by transwell assay (scale bars: 100 μm). (J, K) Cell migration capacity was assessed through wound healing analysis. **p < 0.01, ***p < 0.001.

The transfection efficiency of the transfected cells was verified by qPCR (Figure 3A), and the results showed that sh‐circFUT8 was significantly knocked down in A549 and H1299, and the down‐regulation of circFUT8 in lung cancer cells A549 and H1299 was successfully achieved. CCK8 solution was added at 0, 24, 48 and 72 h, and the absorbance at OD 450 nm was measured in a microplate reader. Each group of cells at each time point was at least three secondary wells. According to the obtained absorbance, the results showed that the viability of circFUT8 knockdown A549 and H1299 cells was significantly lower than that of A549 and H1299 negative control cells (Figure 3B,C). To explore the effects of circFUT8 on cell proliferation, invasion, migration and apoptosis, we first performed cell cloning and counted A549‐NC, A549‐sh‐circFUT8, H1299‐NC, and H1299‐sh‐circFUT8. Five‐hundred cells were seeded in each well in a six‐well plate, and each group of cells had three multiple wells. After cell inoculation, we added an appropriate amount of complete medium to the cells and placed them in a cell incubator to maintain suitable growth conditions. On the following day, we examined the morphology and growth of the cells by microscopy. Based on the observations, we replaced the fresh medium to support continued cell growth and development. Until 1 week of growth, the cells in six‐well plates were fixed and stained, and the results of crystal violet staining showed that sh‐circFUT8 significantly inhibited cell proliferation (Figure 3D,E). The apoptosis of the four groups of cells was detected, and the results of the four groups of cells added with PI and AnnexinV were detected by flow cytometry. The results showed that the apoptosis of A549 and H1299 cells was significantly promoted by knocking down circFUT8 (Figure 3F,G). Cell invasion assay, using the medium concentration difference between the chamber and the culture dish, and then to determine the cell invasion ability. By detecting the cells planted in the chamber for 24, 36, and 48 h, the cells were fixed and stained, and the cells at the bottom of the chamber membrane were observed under the microscope. Our results showed that sh‐circFUT8 inhibited the invasion of A549 and H1299 cells (Figure 3H,I). Four groups of cells in logarithmic phase were selected again and seeded uniformly in six‐well plates, and each group of cells was set up with three multiple wells. After a certain number of cells were planted in each well, the next day when the cell density reached 90% and adhered uniformly to the wall, we used a sterile 200 μL gun tip for cell scratch, and rinsed with PBS to remove floating cell debris. Subsequently, the cells were replaced with 1% FBS + RPMI medium to continue to culture, and the same part was photographed at 0 and 36 h, respectively. The results showed that circFUT8 knockdown inhibited the migration of A549 and H1299 cells (Figure 3J,K).

### 3.4 CircFUT8 Knockdown Inhibits Macrophage M2 Polarization and TNF Signaling Pathway

**FIGURE 4:**
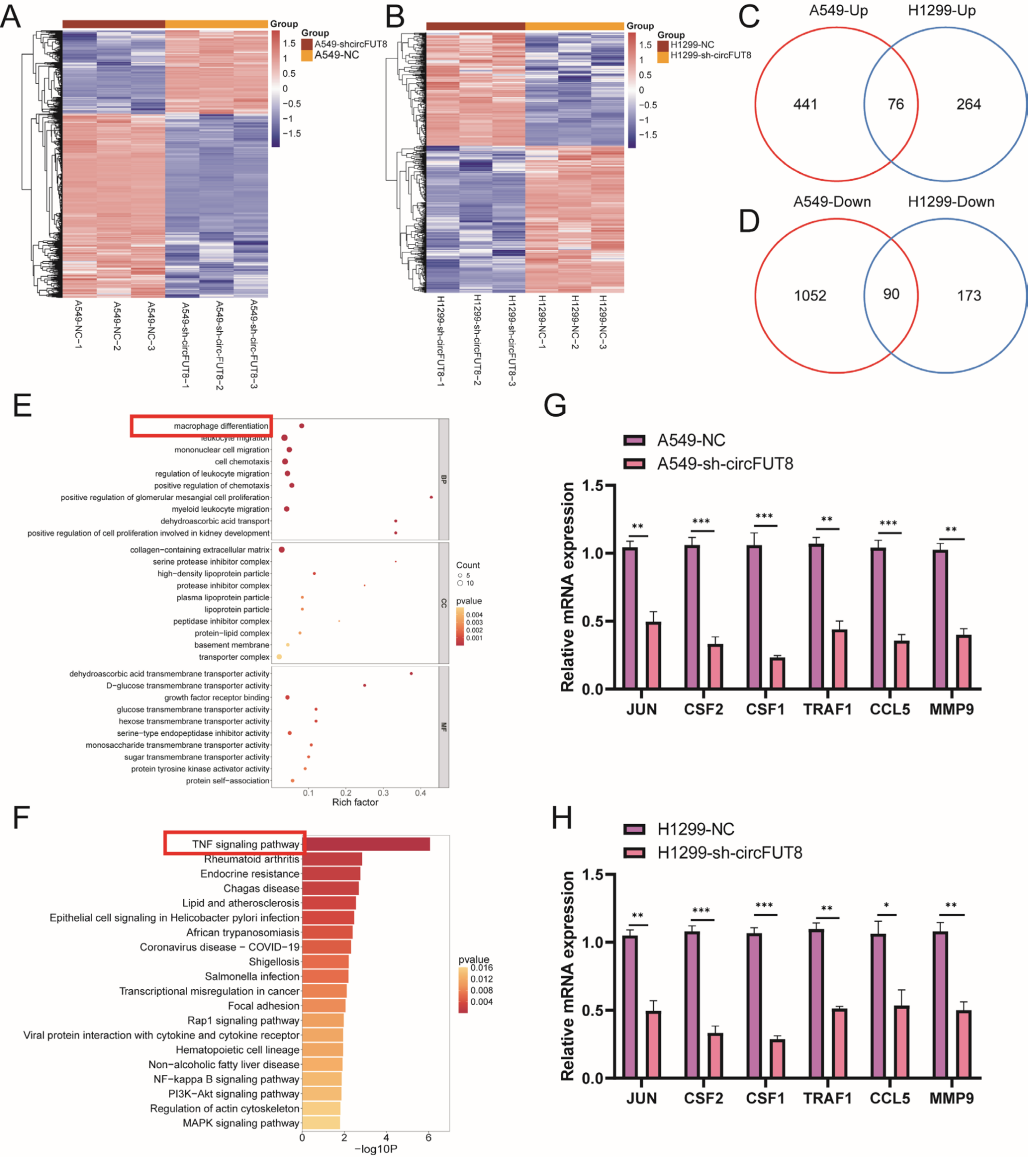
circFUT8 knockdown regulates macrophage differentiation and inhibits the TNF signaling pathway. (A) Heatmap showing differential gene expression profiles in A549 cells with circFUT8 upregulation (sh‐circFUT8) compared to control (NC), as analyzed by RNA‐seq. (B) Heatmap showing differential gene expression profiles in H1299 cells with circFUT8 upregulation (sh‐circFUT8) compared to control (NC), as analyzed by RNA‐seq. (C) Venn diagram comparing upregulated genes in A549 and H1299 cells upon circFUT8 knockdown, highlighting 76 common genes. (D) Venn diagram comparing downregulated genes in A549 and H1299 cells upon circFUT8 knockdown, highlighting 90 common genes. (E) GO enrichment analysis of differentially expressed genes, indicating significant enrichment in pathways related to macrophage differentiation and TNF signaling. (F) KEGG pathway enrichment analysis of differentially expressed genes, showing significant enrichment in the TNF signaling pathway. (G) Quantitative RT‐PCR validation of selected TNF signaling pathway‐related genes (JUN, CSF2, CSF1, TRAF1, CCL5, MMP9) in A549 cells with circFUT8 knockdown compared to control. (H) Quantitative RT‐PCR validation of selected TNF signaling pathway‐related genes (JUN, CSF2, CSF1, TRAF1, CCL5, MMP9) in H1299 cells with circFUT8 knockdown compared to control. *p < 0.05, **p < 0.01, ***p < 0.001.

To further explore the downstream pathways and functions regulated by circFUT8, we used RNA‐seq to detect the functional enrichment and pathway changes of downstream genes regulated by circFUT8 silencing in A549 and H1299 cells. As shown in Figure 4A,B, Heatmap analysis showed many differentially expressed genes in A549 and H1299 cells with circFUT8 silenced compared to the negative control (NC). Venn diagram analysis revealed the number of genes commonly upregulated and downregulated after circFUT8 silencing in A549 and H1299 cells. Results showed 76 genes upregulated and 90 genes downregulated in both cell lines (Figure 4C,D). GO enrichment analysis indicated that DEGs mainly involved macrophage differentiation and regulation of the TNF signaling pathway (Figure 4E). This suggests that circFUT8 may regulate the behavior of lung cancer cells by affecting these biological processes. Bioinformatic analysis through KEGG pathway annotation demonstrated that differentially expressed genes were primarily enriched in the TNF‐related signaling cascade (Figure 4F), indicating that circFUT8 may affect the signal transduction of lung cancer cells by regulating this pathway (Figure S1). Through RT‐qPCR analysis, we found that after silencing circFUT8 in A549 and H1299 cells, the expression levels of TNF signaling pathway‐related genes such as JUN, CSF2, CSF1, TRAF1, CCL5, and MMP9 were significantly decreased (Figure 4G,H). circFUT8 plays an important regulatory role in lung cancer cells by modulating the expression of macrophage differentiation and TNF signaling pathway‐related genes.

### 3.5 CircFUT8 Promotes M2 Macrophage Polarization In Vitro

**FIGURE 5:**
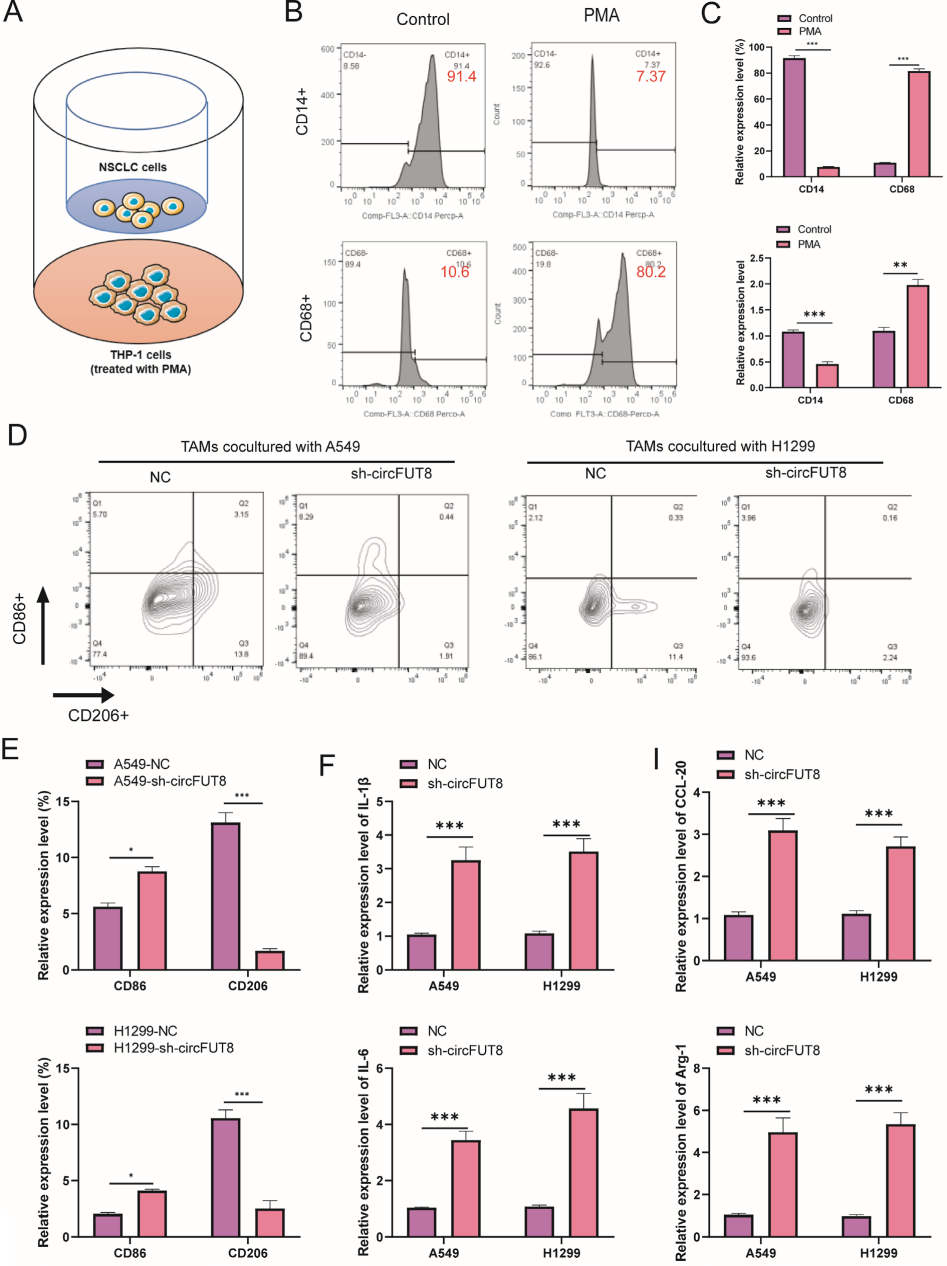
CircFUT8 promoted M2 macrophage polarization. (A) circFUT8 knockdown non‐small cell lung cancer cells were cocultured with PMA‐induced THP‐1 cells. (B) The positive rates of CD14 and CD68 in the co‐culture system of two cell lines (A549 and H1299) were detected by flow cytometry. (C) Quantitative analysis of CD14 and CD68 mRNA expression by RT‐qPCR. (D, E) Flow cytometry was used to detect the M1 macrophage marker CD86 and M2 macrophage marker CD206 in the co‐culture system. (F, G) RT‐qPCR was used to quantitatively analyze M1 macrophage‐associated genes (IL‐1β, CCL‐20, IL‐6) and M2 macrophage‐associated genes (Arg‐1) in the co‐culture system. *p < 0.05, **p < 0.01, ***p < 0.001.

To investigate the functional involvement of circFUT8 in macrophage phenotype regulation, a co‐culture system was established between THP‐1 cells and lung cancer cells transfected with either sh‐circFUT8 or negative control constructs, as illustrated in Figure 5A. Initially, THP‐1 monocytic cells were differentiated into CD68+ M0 macrophages through PMA stimulation at a concentration of 100 ng/mL. The differentiation efficiency was subsequently verified by measuring CD14 and CD68 expression levels using quantitative reverse transcription PCR and flow cytometric analysis. The expression of CD14 and CD68 in the PMA treated group was significantly higher than that in the control group, indicating that PMA induced polarization of THP‐1 cells (Figure 5B,C). Subsequently, flow cytometric analysis was performed to quantify the expression levels of CD86 and CD206, which respectively serve as specific surface markers for M1 and M2 macrophage subtypes, in TAMs derived from the lung cancer co‐culture system (Figure 5D). Our findings indicated that silencing circFUT8 increased the number of CD68‐positive cells while decreasing CD206‐positive cells, as shown in Figure 5E. Consistent with expectations, the knockdown of circFUT8 upregulated markers associated with M1 macrophages (IL‐6 and IL‐1β), as illustrated in Figure 5F, and downregulated markers linked to M2 macrophages (CCL20 and Arg‐1), as demonstrated in Figure 5G. Taken together, circFUT8 in lung cancer cells promoted the polarization of macrophages to M2.

### 3.6 circFUT8 Facilitates Tumor Progression in Lung Cancer Through Inducing M2 Phenotype Transition

**FIGURE 6:**
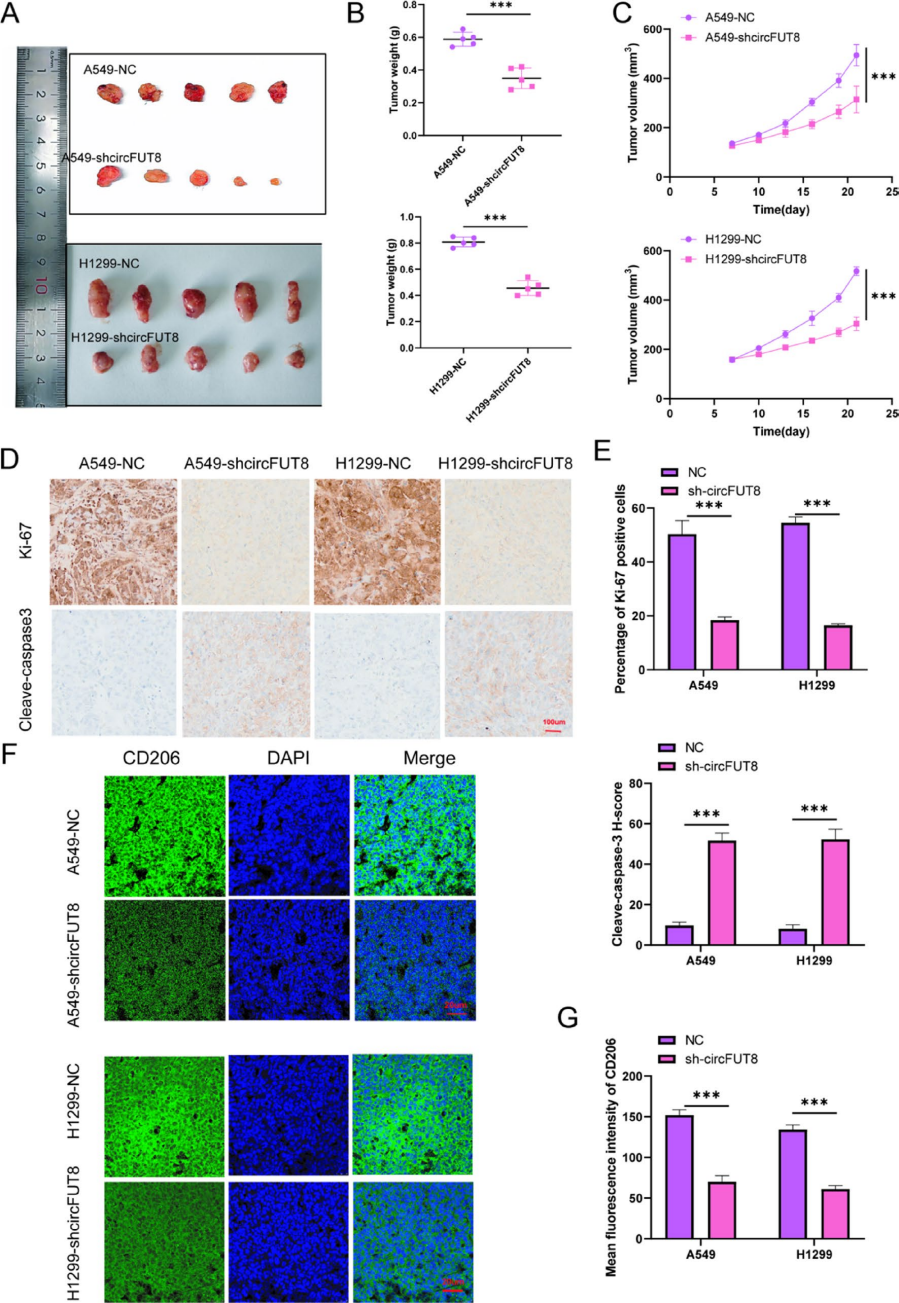
Knockdown of circFUT8 inhibits tumor growth and M2 macrophage polarization while promoting apoptosis. (A) Subcutaneous tumor formation assay in nude mice showing that knockdown of circFUT8 (sh‐circFUT8) significantly inhibits tumor growth. The upper panel displays images of tumors derived from A549 and H1299 cell lines; the lower panel shows a comparison of tumor weights. (B) Quantitative analysis of tumor weights. Tumors with circFUT8 knockdown weighed significantly less than those in the control group (NC). (C) Tumor volume curves over time. The increase in tumor volume was significantly slower in the circFUT8 knockdown group compared to the control group. (D) Immunohistochemical (IHC) staining indicating that knockdown of circFUT8 increases the expression of the apoptosis marker cleaved caspase‐3 and decreases Ki‐67 protein (scale bars: 100 μm). (E) Quantitative analysis of the Ki‐67 labeling index and Cleaved‐caspase3 H‐score from IHC staining (*n* = 5 tumors per group). (F) Immunofluorescence (IF) staining showing that knockdown of circFUT8 reduces the expression of CD206. Nuclei are stained with DAPI, and scale bars: 20 μm. (G) Quantitative analysis of CD206 mean fluorescence intensity (MFI) from IF staining (*n* = 5 tumors per group, 5 fields per tumor). Data are presented as mean ± SD. ***p < 0.001.

To evaluate the biological role of circFUT8 under physiological conditions, xenograft models were generated by subcutaneously implanting both control and circFUT8‐knockdown A549/H1299 cells into immunodeficient mice (Figure 6A). Tumor dimensions were monitored at three‐day intervals throughout the study period, with final tumor mass quantification performed upon experimental termination. Compared to the NC group, the tumor weight significantly decreased after silencing circFUT8 (Figure 6B), and the tumor growth curve showed that the tumor volume increased more slowly after silencing circFUT8 (Figure 6C). The expression patterns of cellular proliferation and apoptosis markers were analyzed through immunohistochemical analysis, utilizing specific antibodies against Ki‐67 and cleaved caspase‐3, respectively. Quantitative immunohistochemical analysis revealed that silencing circFUT8 significantly reduced the Ki‐67 labeling index and increased the H‐score of cleaved caspase‐3 in xenograft tumor tissues (Figure 6D,E). Consistent with this, immunofluorescence analysis demonstrated a significant reduction in the MFI of the M2 macrophage marker CD206 in the sh‐circFUT8 group compared to the NC group, indicating that silencing circFUT8 inhibited M2 macrophage polarization in vivo (Figure 6F,G). circFUT8 plays an important role in tumor formation in lung cancer, and its silencing can inhibit tumor growth and polarization of M2 type macrophages.

### 3.7 CircFUT8 Interacts With ENO1 Directly to Promote M2 Macrophage Polarization

**FIGURE 7:**
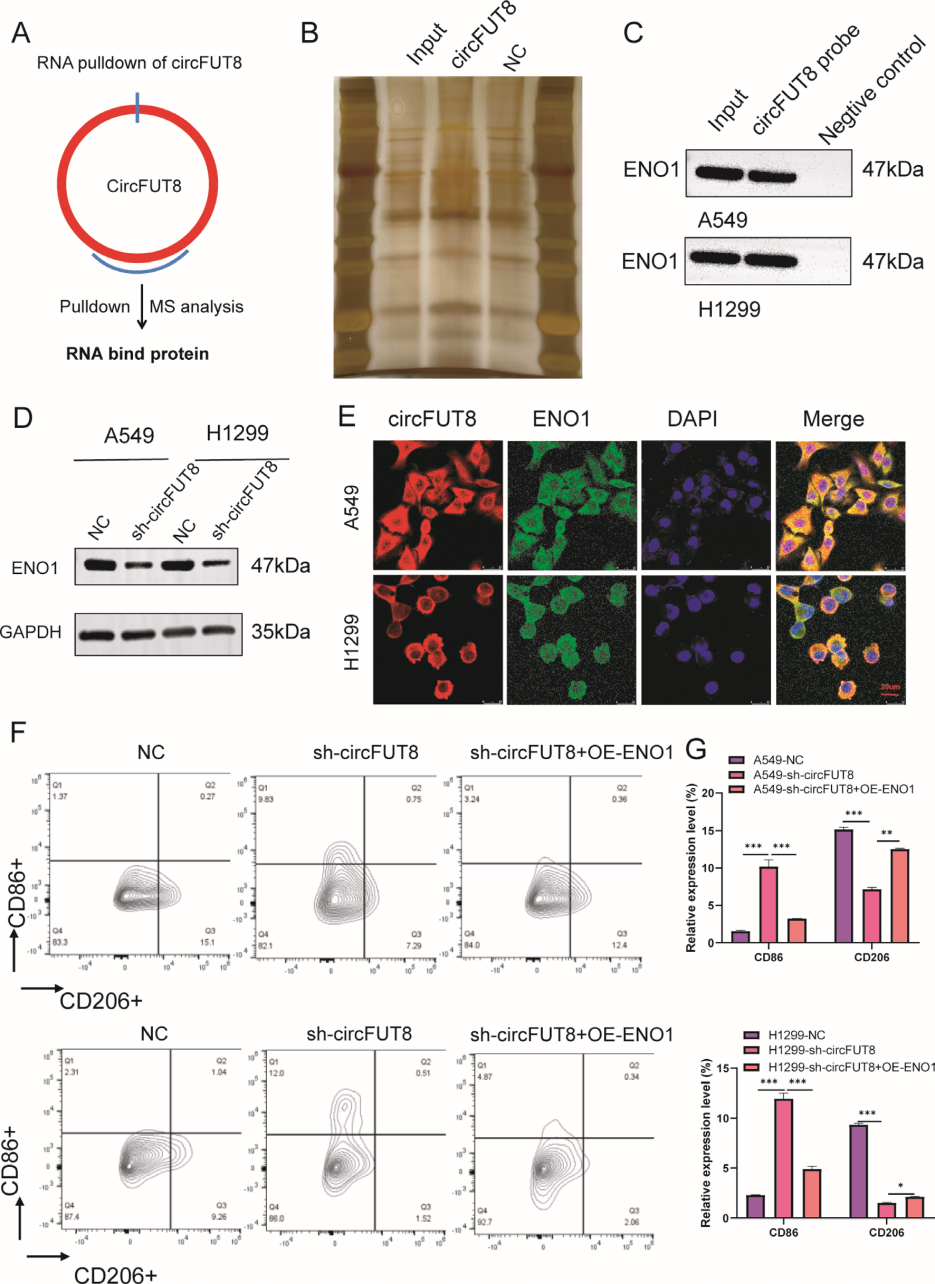
CircFUT8 interacts with ENO1 to promote M2 macrophage polarization. (A) Mass spectrometry identification of circFUT8‐interacting proteins using junction‐specific probes in A549 cell lysates, followed by protein complex characterization. (B) Proteomic profiling of circFUT8‐associated protein complexes isolated through RNA pull‐down assays. (C) RNA‐protein interaction analysis demonstrating specific binding between ENO1 and circFUT8 through immunoblot validation of RNA pull‐down assays. (D) Protein expression analysis of ENO1 in circFUT8‐depleted lung cancer cells compared to controls, with GAPDH serving as loading control. (E) Subcellular colocalization analysis of circFUT8 and ENO1 using combined immunofluorescence and fluorescence in situ hybridization techniques (scale bars: 20 μm). (F, G) Functional rescue experiments assessing macrophage polarization markers (CD86 and CD206) through flow cytometry in M0 TAMs co‐cultured with circFUT8‐knockdown lung cancer cells. The percentage of CD86 and CD206 positive cells was examined by flow cytometry. Results are presented as Mean ± SD of a representative experiment performed in triplicate. **p < 0.01, ***p < 0.001.

To elucidate the molecular basis underlying circFUT8‐mediated TAM M2 phenotype transition, RNA‐protein interaction profiling was performed using biotin‐labeled circFUT8 probes coupled with mass spectrometry identification, with control probes serving as negative references (Figure 7A,B). Through this screening approach, ENO1 emerged as a candidate binding partner that physically interacts with circFUT8 (Figure S2), which is known to play critical roles in protein homeostasis maintenance (Figure 7C). Subsequent immunoblotting analysis validated that circFUT8 knockdown significantly decreased ENO1 expression levels in both A549 and H1299 cell lines (Figure 7D). Spatial distribution analysis using combined immunofluorescence and fluorescence in situ hybridization techniques revealed cytoplasmic colocalization of circFUT8 and ENO1 (Figure 7E), suggesting the formation of functional RNA‐protein complexes in this cellular compartment.

To further investigate the circFUT8/ENO1 axis in macrophage polarization regulation, a rescue experiment was designed where THP‐1 cells were cocultured with NSCLC cells subjected to simultaneous circFUT8 knockdown and ENO1 overexpression. Flow cytometric analysis demonstrated that while circFUT8 depletion increased the CD68+ cell population and decreased CD206+ cells, these effects were counteracted by ENO1 upregulation (Figure 7F,G).

### 3.8 ENO1 Promotes TNF Signaling Through Glycolytic Metabolites

**FIGURE 8:**
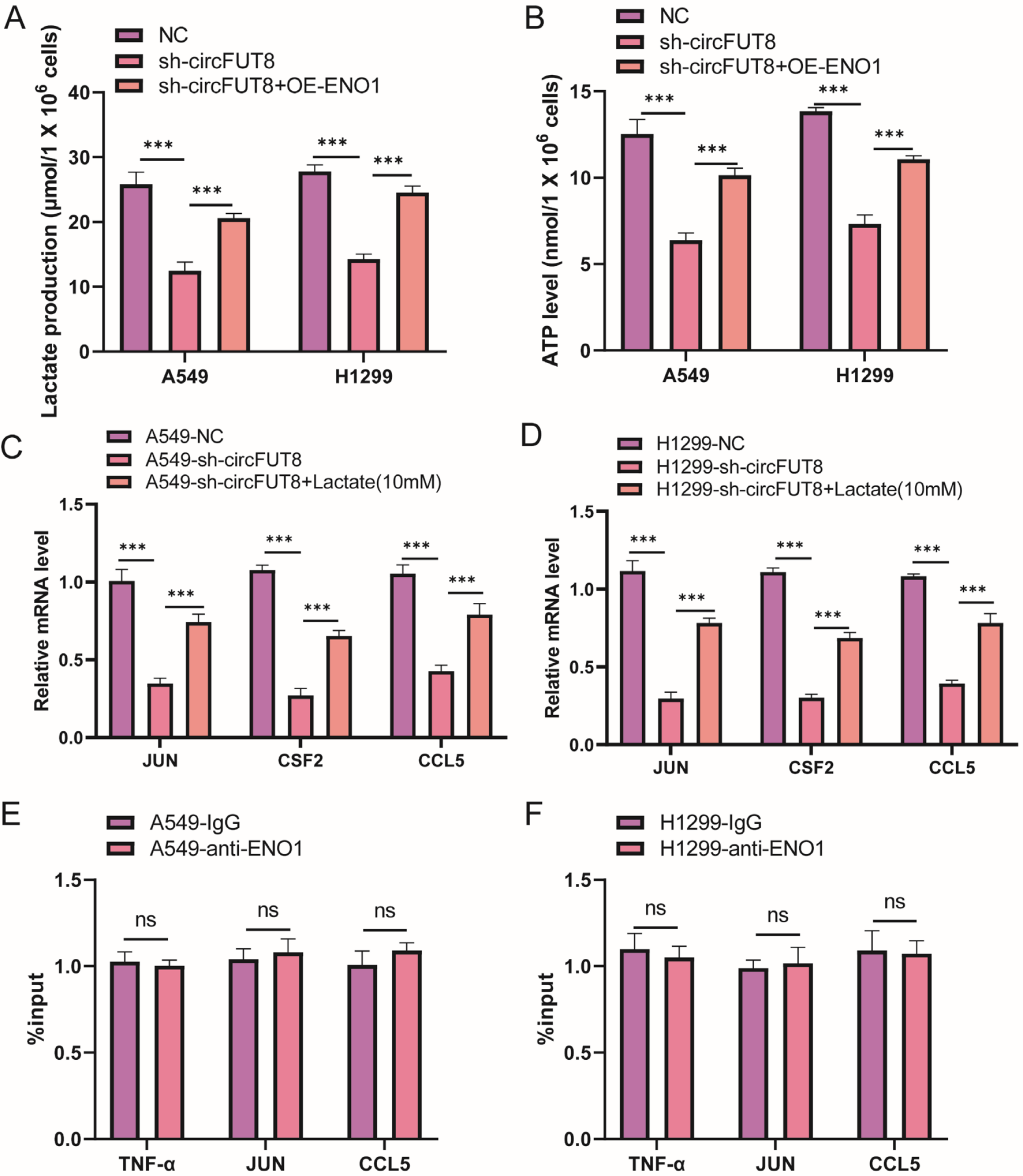
circFUT8 recruits ENO1 to enhance glycolytic lactate production, which activates the TNF signaling pathway. (A) Measurement of lactate production in lung cancer cells under the indicated conditions. circFUT8 knockdown impairs glycolysis, which is rescued by ENO1 overexpression. (B) Measurement of intracellular ATP levels in lung cancer cells under the indicated conditions. (C) qRT‐PCR analysis of TNF signaling pathway gene expression (JUN, CSF2, CCL5) in A549 cells. (D) qRT‐PCR analysis of TNF signaling pathway gene expression (JUN, CSF2, CCL5) in H1299 cells. (E) ChIP‐qPCR analysis showing the lack of significant enrichment of ENO1 at the promoter regions of TNF pathway genes compared to the IgG control in A549 cells. (F) ChIP‐qPCR analysis showing the lack of significant enrichment of ENO1 at the promoter regions of TNF pathway genes compared to the IgG control in H1299 cells. A known non‐target region (GAPDH promoter) serves as a negative control. Data are presented as mean ± SD from at least three independent experiments. *p < 0.05, **p < 0.01, ***p < 0.001; ns, not significant (one‐way ANOVA with Tukey's post hoc test).

To investigate whether ENO1 regulates TNF signaling via its glycolytic activity, we measured lactate and ATP levels in the culture supernatant of A549 and H1299 cells upon circFUT8 knockdown/overexpression of ENO1. We found that silencing circFUT8 significantly reduced lactate production and ATP levels, which were rescued by ENO1 overexpression (Figure 8A,B). Furthermore, exogenous lactate supplementation partially restored the expression of TNF pathway genes (JUN, CSF2, CCL5) in circFUT8 knockdown cells (Figure 8C,D). These results suggest that ENO1‐derived lactate may serve as a key metabolite linking glycolytic activity to TNF signaling activation.

To examine whether ENO1 acts as a transcriptional regulator, we performed chromatin immunoprecipitation (ChIP) assay using an anti‐ENO1 antibody. We did not observe significant enrichment of ENO1 at the promoter regions of TNF, JUN, CCL5 (Figure 8E,F), suggesting that ENO1 regulates TNF signaling indirectly, likely through metabolic reprogramming rather than direct transcriptional control.

The TNF signaling pathway and these differential genes were analyzed by protein interaction analysis, KEGG pathway enrichment analysis and GO functional annotation enrichment analysis. Through KEGG, we found that these differential gene biological signaling pathway network involves COVID‐19, NF‐kappa B, TNF, PI3K‐AkT, cytokines and their receptors and other signaling pathways. The enrichment of differential genes in the KEGG COVID‐19 pathway suggested their possible involvement in viral invasion (such as HBEGF enhancing viral endocytosis through EGFR signaling) or host immune response (such as CXCR4‐mediated regulation of cytokine storm). The TNF signaling pathway plays a key role in regulating inflammatory response, cell proliferation, apoptosis and immune response. Activation of genes related to this pathway (CSF2, SERPINE1, PDGFD, CYFIP2, IL7R, MYB, CSF1) can promote tumor cell proliferation and survival, and may also affect immune cells in the TME. Thus, it affects tumor progression and treatment response. The PI3K‐Akt signaling pathway plays a key role in cell growth, survival, metabolism, and motility, and activation of its related genes (MYD88, IL7R, MYB, CSF1) has been implicated in the initiation and progression of several cancers (Figure S1A). GO function analysis revealed that MMP9, SAA1, and TRIM55 were related to monocyte migration, CSF2, CSF1, and HBEGF were related to macrophage polarization, and CCL5, PLA2G7, and RAC2 may be involved in the regulation of leukocyte migration. It affects the localization and function of immune cells (Figure S1B).

By mass spectrometry, we found that ENO1 ranked second in the list of proteinscircFUT8 interacting protein (Figure S2A). ENO1 was successfully identified by RNA pull‐down assay and mass spectrometry. The peptide coverage map of ENO1 protein, in which the blue‐marked regions represent the detected peptides, and the coverage of these peptides indicates a significant interaction between ENO1 and circFUT8 (Figure S2C). The mass spectrogram of a unique peptide in ENO1 protein is further shown, and the high‐quality match of the mass spectrometric data with the specific peptide sequence indicates that the peptide was identified with high confidence (Figure S2D).

## 4 Discussion

Lung cancer for significant cancer‐related morbidity and mortality worldwide [33, 34, 35]. The RNase R stable characteristics of CircRNAs shows promising potential in cancer diagnostics and prognostics prediction [36, 37]. Emerging evidence indicates that circRNA molecules serve as potential diagnostic and prognostic biomarkers in lung cancer and other cell types [16, 36, 38]. In this study, hsa_circ_0003028 (designated as circFUT8) demonstrated significant upregulation in lung cancer tissues and showed strong correlation with unfavorable clinical outcomes. Our investigation revealed a significant correlation between elevated hsa_circ_0003028 levels in lung cancer specimens and advanced tumor size as well as poorer clinical staging.

In lung cancer, CircRNAs are emerging as oncodrivers/tumor suppressors and promising biomarkers [9, 13, 14], by regulating cell proliferation, apoptosis, invasion, and metastasis, thereby influencing the progression of lung cancer [15, 16]. Recent studies show that inhibiting the malignant behavior of cancer cells shows promising potential in lung cancer therapeutics [39, 40, 41]. In this study, functional studies revealed that circFUT8 increased cellular proliferation, migratory capacity, and invasive potential, while simultaneously suppressing programmed cell death. Cell death resistance and metabolism reprogramming are two pivotal hallmarks in cancer [42]. Tumor cells often resist apoptosis through metabolic reprogramming, particularly by enhancing glycolysis. The Warburg effect allows cancer cells to generate ATP rapidly to support proliferation and survival under hypoxic conditions [43, 44]. This study identified circFUT8 as a CircRNA link between cell death resistance and possibly regulating glycolysis in lung cancer.

In addition to modulating tumor cell malignancies, we also found that circFUT8 induces an immuno suppressive microenvironment. Our results demonstrated that circFUT8 up‐regulated the expression and secretion of CSF1 and CCL5 by activating TNF‐⍺ signaling. This activation also induced M2 macrophage polarization, suggesting that circFUT8 may serve as a potential therapeutic target for the treatment of lung cancer. More importantly, we found that circFUT8 facilitates the M2 polarization of TAMs. TAMs serve as key immunosuppressive regulators influencing cancer progression [21]. Studies shows that circRNAs regulate TAM polarization in cancer progression [21, 27, 45, 46]. For example, circITGB6 promotes ovarian cancer resistance by shifting TAMs toward the M2 phenotype [47]. CircATP9A in NSCLC enhances progression through M2 macrophage polarization [21]. In colorectal cancer, targeting circ‐0034880 in tumor exosomes inhibits pre‐metastatic niche formation driven by pro‐tumor macrophages [25]. This study identified a glycolysis‐regulating oncogenic role of circFUT8 in lung cancer progression and microenvironment reprogramming.

CircRNAs possess diverse functional capacities, such as sequestering miRNAs, modulating transcriptional initiation and elongation, recruiting epigenetic modifiers, and regulating alternative splicing processes [9, 10, 48, 49]. In this study, RNA Pulldown was used to find proteins that might interact with hsa_circ_0003028. Mass spectrometry analysis provided evidence that hsa_circ_0003028 interacted with RNA‐binding protein ENO1 (a‐enolase) and regulated the biological function of macrophages. Enolase 1 (ENO1) is a glycolytic enzyme that plays a crucial role in cancer progression via cell death regulation [50], epithelial‐to‐mesenchymal transition [51], and metabolism reprogramming [52, 53, 54]. Additionally, ENO1 promotes the M2 polarization of TAMs through glycolysis‐dependent and independent mechanisms, which supports tumor progression [55, 56]. The M2 polarization of TAMs may be related to the glycolysis process to generate energy substances such as ATP and metabolites to supply energy for cells [57, 58, 59]. Our findings are consistent with previous studies and introduce new evidence that circRNAs interact with RNA‐binding proteins to regulate the expression of target genes.

Our study further elucidates that ENO1, upon binding to circFUT8, enhances glycolytic flux, leading to increased lactate production. This lactate‐rich microenvironment subsequently activates TNF signaling in a paracrine manner, promoting M2 macrophage polarization. Although ENO1 has been reported to possess transcriptional co‐activator functions in certain contexts, our ChIP data did not support direct DNA binding, suggesting that its role here is primarily metabolic.

While our study provides direct evidence that the circFUT8‐ENO1 axis drives M2 macrophage polarization, the immunosuppressive TME is a complex ecosystem comprising various immune cell types. It is well established that M2‐polarized TAMs do not operate in isolation but actively engage in crosstalk with other immune constituents. For instance, M2 macrophages are known to suppress cytotoxic CD8+ T cell function through the release of anti‐inflammatory cytokines like IL‐10 and TGF‐β, and to recruit regulatory T cells (Tregs) and neutrophils via chemokines such as CCL17, CCL22, and CCL5 [60]. Notably, several of these factors (e.g., CCL5) were identified as downstream targets of circFUT8 in our RNA‐seq data. Therefore, although beyond the scope of this current study, it is plausible that circFUT8‐driven M2 polarization indirectly contributes to a broader immunosuppressive network by impairing T cell immunity and promoting the recruitment of pro‐tumor neutrophils and Tregs. Future research should explicitly investigate these potential interactions in an immunocompetent setting.

Beyond elucidating a novel oncogenic mechanism, our findings unveil significant translational potential for circFUT8 in lung cancer management. Firstly, the pronounced upregulation of circFUT8 in tumor tissues and its strong association with advanced stage and poor prognosis position it as a promising diagnostic and prognostic biomarker. Given the high stability of circRNAs in body fluids [61], detecting circFUT8 in plasma or serum exosomes via liquid biopsy presents a compelling non‐invasive strategy for early detection, patient stratification, and monitoring therapeutic response.

More importantly, our study nominates circFUT8 as a compelling therapeutic target. The specificity of circRNAs, defined by their unique back‐splice junctions, offers a therapeutic window to selectively target cancer cells while minimizing off‐target effects on host linear transcripts. Targeting the resulting immunosuppressive microenvironment, for instance by inhibiting lactate production or its transporters [62], could synergize with current immunotherapies (e.g., anti‐PD‐1/PD‐L1 antibodies) to reactivate antitumor immunity. Future work should focus on developing efficient delivery vehicles for circFUT8‐targeting agents and evaluating their efficacy, both alone and in combination with immunotherapy, in immunocompetent lung cancer models.

Finally, our study highlights the role of circFUT8 in lung cancer, but limitations exist. First, our findings are based on cell and animal models, requiring validation in clinical samples. Second, the detailed mechanism of interaction between circFUT8 and ENO1 remains unclear. Third, while the overall sample size was sufficient for detecting circFUT8 expression differences, subgroup analyses (e.g., by TNM stage) were limited by smaller sample sizes. Future studies with larger cohorts are warranted to validate these associations. Future work should focus on clarifying how glycolysis influences macrophage function in the TME, potentially through measuring indicators like lactic acid, glucose, and ATP. This study strengthens the evidence that hsa_circ_0003028 promotes LUAD progression via TAM M2 polarization by binding to ENO1, suggesting it as a potential therapeutic target. Further exploration of circFUT8's role in other cancers is also needed.

## 5 Conclusions

In conclusion, our findings highlight the significant role of circFUT8 in the progression of lung cancer, particularly in promoting M2 macrophage polarization. The interaction between circFUT8 and ENO1 represents a novel therapeutic target for lung cancer, and future research will further investigate the potential applications of circFUT8 in cancer therapy.

## Author Contributions

**Yang Ren:** conceptualization, methodology, investigation, formal analysis, writing – original draft. **Yidan Shen:** methodology, validation, investigation, data curation. **Qingguo Wu:** software, validation, visualization. **Peng Zhang:** investigation, resources, data curation. **Lei Wang:** investigation, formal analysis, visualization. **Feng Li:** conceptualization, writing – review and editing, supervision. **Yinzhong Shen:** conceptualization, writing – review and editing, supervision, project administration, funding acquisition. All authors read and approved the final manuscript.

## Funding

This work was supported by the Shanghai hospital development center (SHDC22024317) and Shanghai Public Health Clinical Center (KY‐GW‐2021‐15).

## Ethics Statement

The experiment protocol listed below has been reviewed and approved by Shanghai Public Health Clinical Center Ethics Committee Approval Letter (No. 2020‐Y076‐01).

## Conflicts of Interest

The authors declare no conflicts of interest.

## Supporting information


**Figure S1:** Gene function annotation and pathway enrichment analysis of circFUT8 knockdown model. (A) Analysis of the biological signaling pathway network by KEGG enrichment. (B) further discovery of enrichment of molecular processes by GO function.
**Figure S2:** RNA pull‐down and MS analysis identified that CircFUT8 interacted with ENO1. (A) In the list of differential proteins detected by mass spectrometry, ENO1 was in the second place. (B) The peptide coverage map of ENO1 protein, in which the blue‐marked regions represent the detected peptides, and the coverage of these peptides indicates a significant interaction between ENO1 and circFUT8. (C) Mass spectrogram of a unique peptide in ENO1 protein, and the high quality of the mass spectrometric data matched the specific peptide sequence, indicating that the peptide was identified with high confidence.

## Data Availability

The data that support the findings of this study are available from the corresponding author upon reasonable request.
