# Supplementary material for: Fucosyltransferase 8‐Derived Circular RNA Drives M2 Polarization of Macrophages Through ENO1‐TNF Signaling Axis to Promote Lung Cancer Progression
Source: Thorac Cancer. 2025 Dec 2;16(23):e70194. doi: 10.1111/1759-7714.70194 (PMC12670297; doi:10.1111/1759-7714.70194)
Supplement: Supplementary file 1 — Figure S1: Gene function annotation and pathway enrichment analysis of circFUT8 knockdown model. (A) Analysis of the biological signaling pathway network by KEGG enrichment. (B) further discovery of enrichment of molecular processes by GO function. Figure S2: RNA pull‐down and MS analysis identified that CircFUT8 interacted with ENO1. (A) In the list of differential proteins detected by mass spectrometry, ENO1 was in the second place. (B) The peptide coverage map of ENO1 protein, in which the blue‐marked regions represent the detected peptides, and the coverage of these peptides indicates a significant interaction between ENO1 and circFUT8. (C) Mass spectrogram of a unique peptide in ENO1 protein, and the high quality of the mass spectrometric data matched the specific peptide sequence, indicating that the peptide was identified with high confidence. [file TCA-16-e70194-s001.docx]

**Supplementary Figure Legends**

**
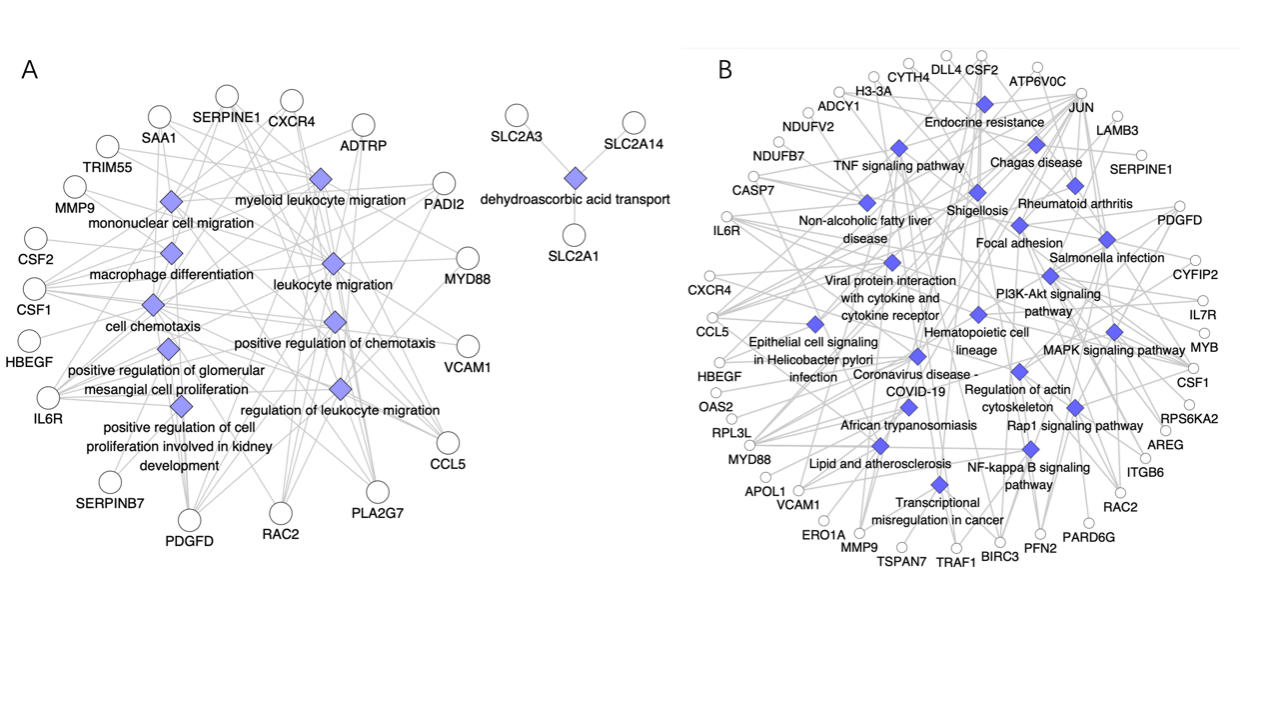
Figure S1 Gene function annotation and pathway enrichment analysis of circFUT8 knockdown model**

(A) Analysis of the biological signaling pathway network by KEGG enrichment.

(B) further discovery of enrichment of molecular processes by GO function.


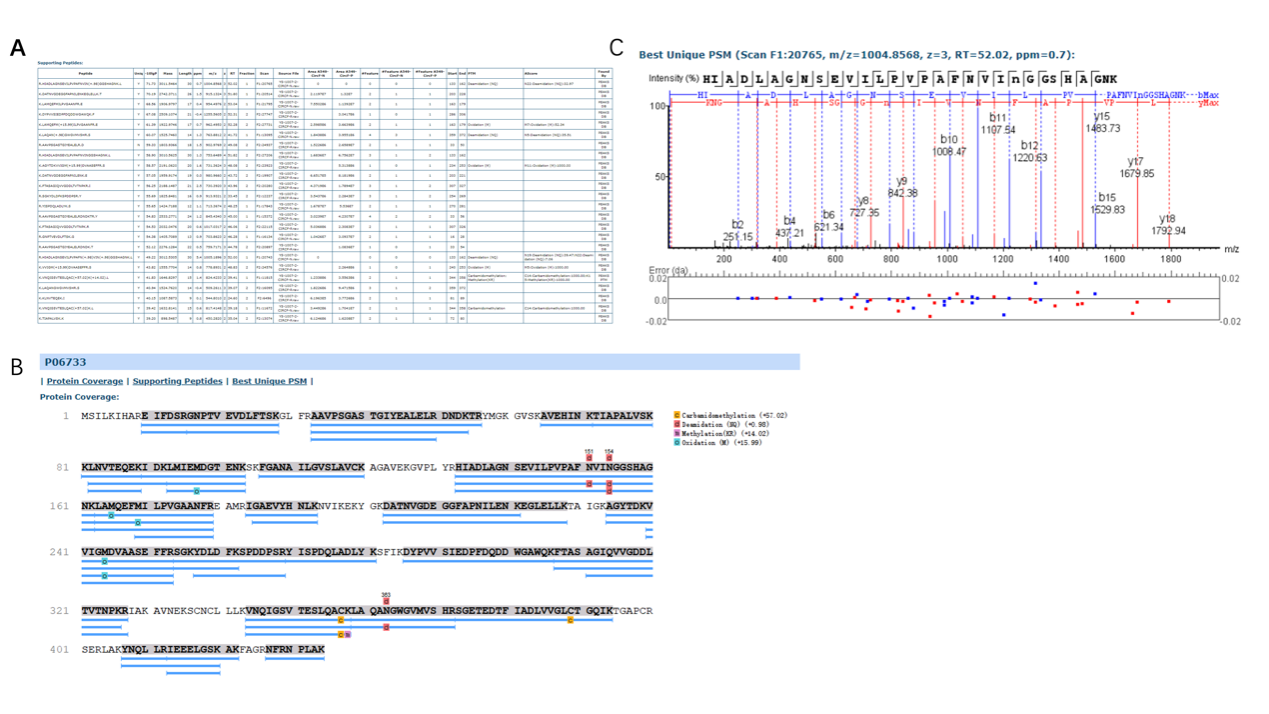
**Figure S2 RNA pull-down and MS analysis identified that CircFUT8 interacted with ENO1**

(A) In the list of differential proteins detected by mass spectrometry, ENO1 was in the second place.

(B) The peptide coverage map of ENO1 protein, in which the blue marked regions represent the detected peptides, and the coverage of these peptides indicates a significant interaction between ENO1 and circFUT8;

(C) Mass spectrogram of a unique peptide in ENO1 protein, and the high quality of the mass spectrometric data matched the specific peptide sequence, indicating that the peptide was identified with high confidence.
